# Supplementary figures and images for: Pervasive antagonistic interactions among hybrid incompatibility loci
Source: PLoS Genet. 2017 Jun 12;13(6):e1006817. doi: 10.1371/journal.pgen.1006817 (PMC5484531; doi:10.1371/journal.pgen.1006817)

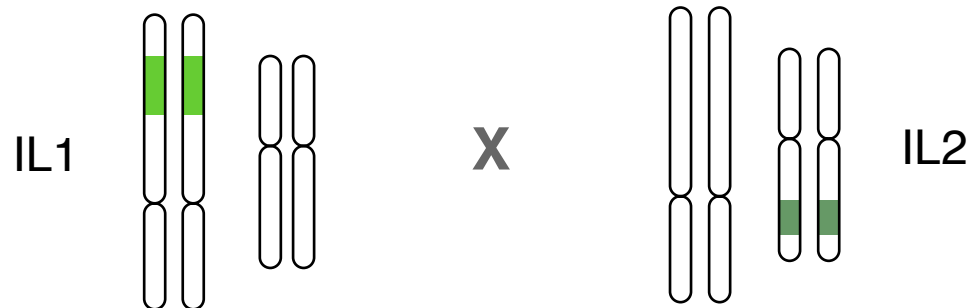

X

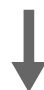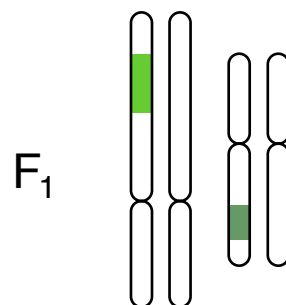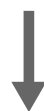

Selfing

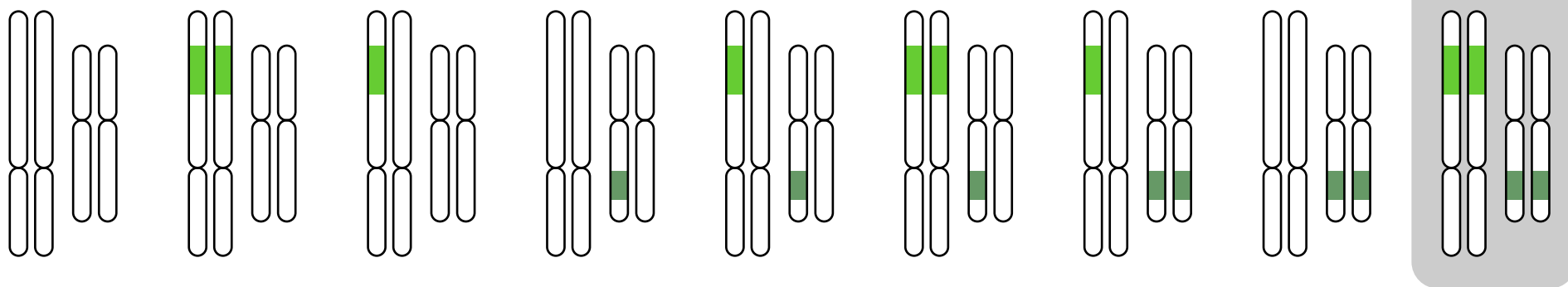

0.0625 : 0.0625 : 0.0125 : 0.0125 : 0.025 : 0.0125 : 0.0125 : 0.0625 : 0.0625

Supplement: S1 Fig — Green shaded areas within a chromosome represent introgressed hab regions in an isogenic lyc background. Included are the expected genotypic ratios in an F2 population for each DIL family, if marker transmission is Mendelian. (PDF) [file pgen.1006817.s005.pdf]

**Uncorrected**

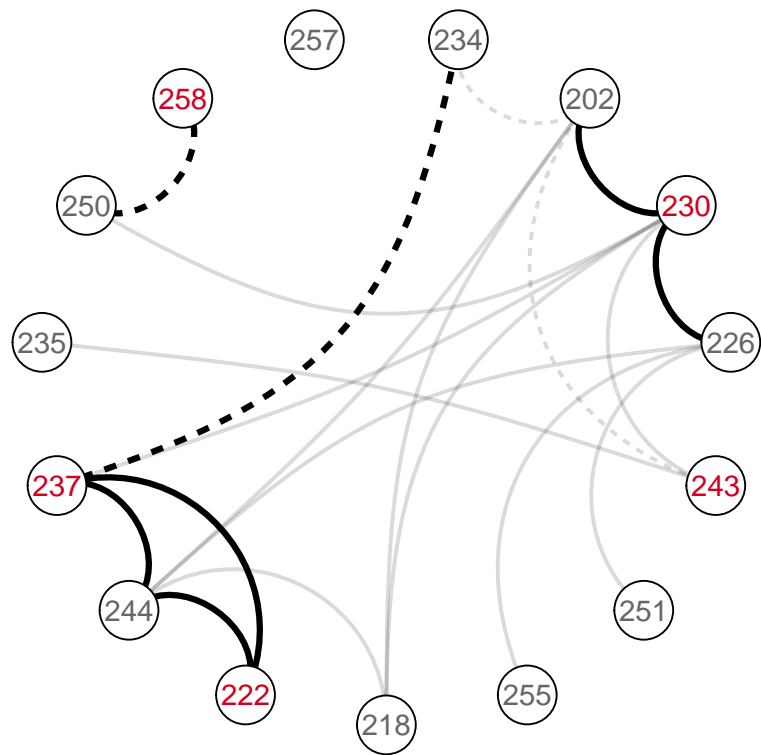

**Pollen-corrected**

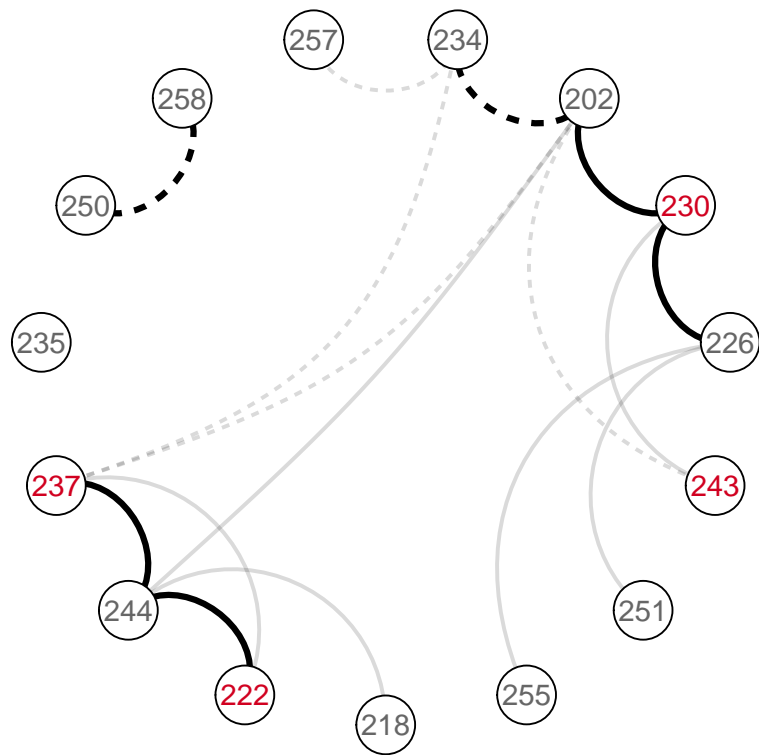

Supplement: S2 Fig — Parental ILs (nodes; red label = sterile IL) are connected if they showed a highly significant interaction (FDR = 1%). Solid lines denote antagonistic interactions; dashed lines mean synergistic interactions. (PDF) [file pgen.1006817.s006.pdf]

Seed

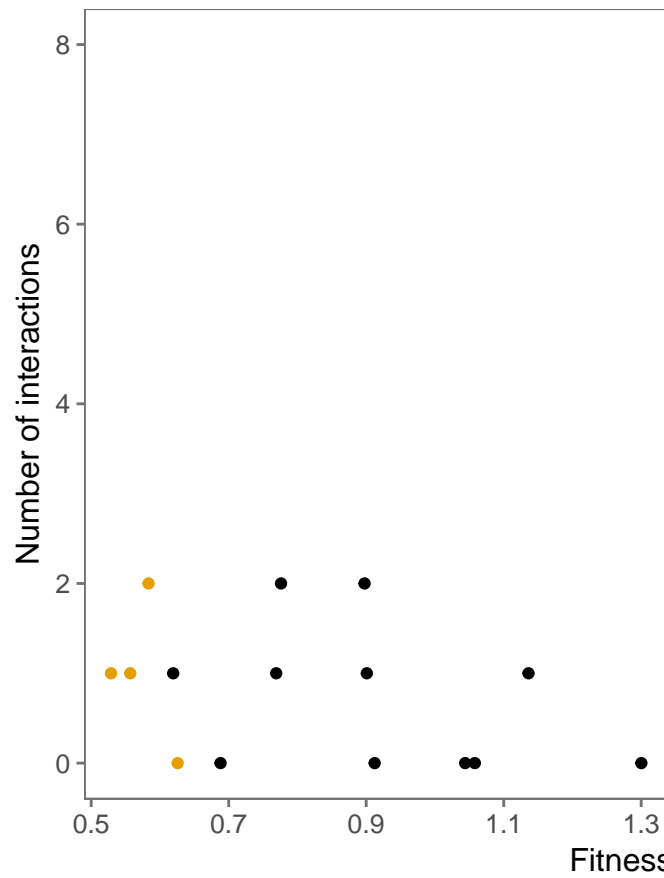

Pollen

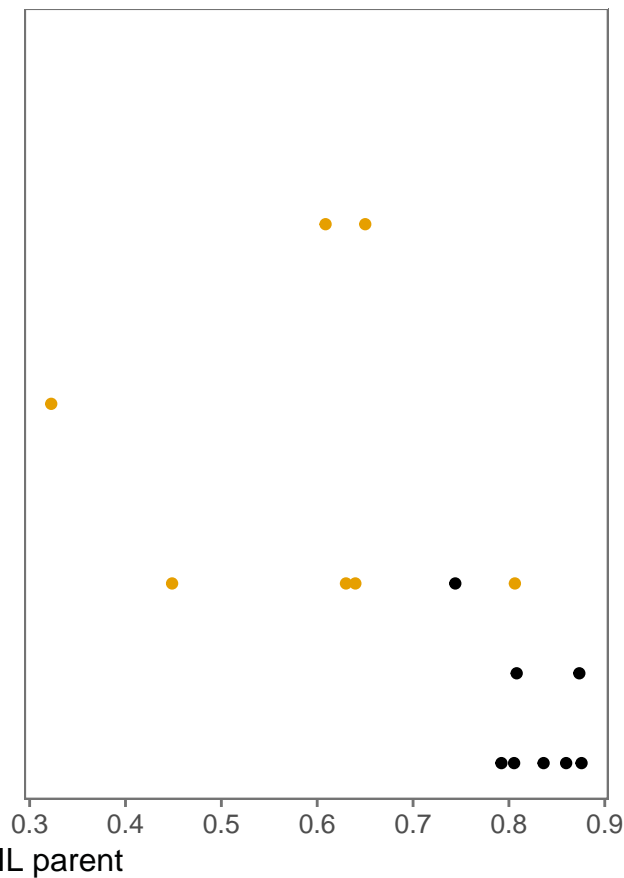

Supplement: S3 Fig — In pollen, ILs with larger effects on fitness tend to show more interactions (Negative-binomial GLM P = 0.003, pseudo-R2 = 0.13). This does not seem to be the case for seed phenotypes. Yellow circles represent sterile ILs, black circles are ILs with no individual effects on fitness (see Table 1). (PDF) [file pgen.1006817.s007.pdf]

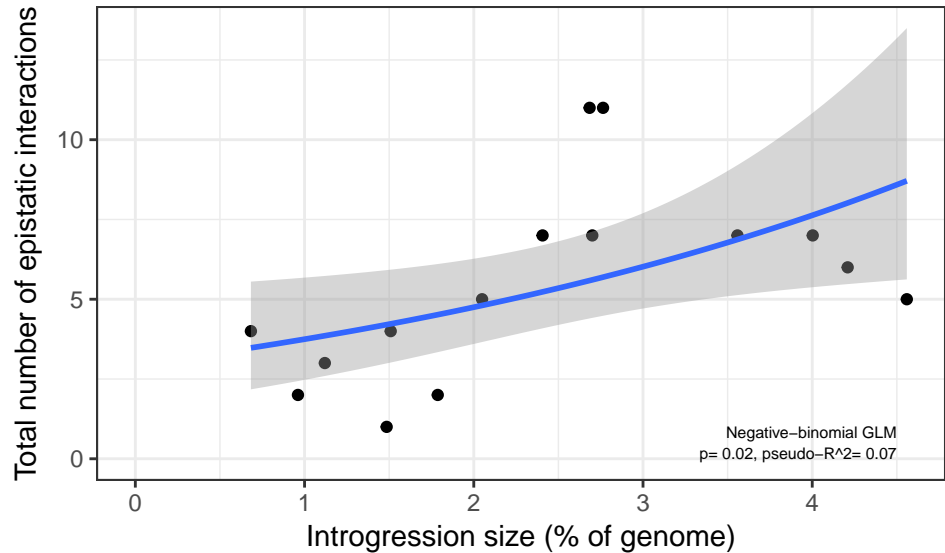

Supplement: S4 Fig — The total number of interactions is the sum of highly significant (FDR 1%) interactions observed in pollen and seed fertility. (PDF) [file pgen.1006817.s008.pdf]
